# Intra-Host Evolution of Norovirus GII.4 in a Chronic Infected Patient With Hematopoietic Stem Cell Transplantation

**DOI:** 10.3389/fmicb.2020.00375

**Published:** 2020-03-09

**Authors:** Jie-mei Yu, Ze-yin Liang, Ke Guo, Xiao-man Sun, Qing Zhang, Yu-jun Dong, Zhao-jun Duan

**Affiliations:** ^1^College of Life Sciences and Bioengineering, Beijing Jiaotong University, Beijing, China; ^2^National Institute for Viral Disease Control and Prevention, Chinese Center for Disease Control and Prevention, Beijing, China; ^3^Department of Hematology, Peking University First Hospital, Beijing, China

**Keywords:** human norovirus, evolution, chronic infection, intra-host, hematopoietic stem cell transplantation

## Abstract

Human noroviruses (NVs) are the leading cause of acute gastroenteritis outbreaks worldwide. The majority of outbreaks are caused by genogroup II.4 (GII.4), with new variants emerging every 2 to 4 years. Immunocompromised patients are hypothesized to be important reservoirs where new NV variants emerge. Here, we examined intra-host NV variants and assessed immune-driven NV evolution in chronically infected immunocompromised hosts. Three NV GII.4-positive samples were collected from the same patient in different clinical phases following allogeneic hematopoietic stem cell transplantation, and had viral RNA concentrations of 2.46 × 10^6^, 1.47 × 10^6^, and 2.26 × 10^6^ genome copies/mL. The non-synonymous (dN) and synonymous (dS) substitution ratio of the sequences in the partial P domain were >1, indicating strong positive selection in the patient. Both the number and the frequency of the single nucleotide variants increased over time in the patient. Also, the majority of capsid amino acid changes were located at blocking epitopes and histo-blood group antigen (HBGA)-binding sites, and 11 positive selection sites were found in the capsid region, of which 8 sites were presented in blocking epitopes or HBGA-binding sites. Homodimeric P-domain capsid models also suggested a structural change in the epitopes and HBGA-binding sites. The results suggested that novel variants of NV GII.4 with HBGA and antigenic site changes were produced in the immunocompromised patient. Further functional and epidemiological studies are needed to determine whether the new variants are a risk to public health.

## Introduction

Noroviruses (NVs) are the leading pathogens necessitating community and outpatient visits for acute gastroenteritis (Tam et al., 2012), and are the leading cause of gastroenteritis outbreaks worldwide, causing approximately half of all such outbreaks (Glass et al., 2009). Although NV infections typically result in acute and self-limiting gastroenteritis, young children, the elderly people and immunocompromised patients are vulnerable to more severe and prolonged infections. In particular, immuno-incompetent and transplant patients can be chronically infected with NVs. NVs are small, non-enveloped viruses with a genome of 7500 nucleotides (nt) of single positive-stranded RNA. The genome is organized into three open reading frames (ORFs). ORF1 is translated as a polyprotein that is cleaved into six non-structural proteins, while ORF2 and ORF3 encode the major capsid protein (VP1) and minor capsid protein (VP2), respectively.

The NV capsid is formed from 90 VP1 dimers, each of which comprised a shell (S) domain and a protruding (P) domain. The P domain is further divided into the P1 and P2 subdomains. Compared with the S domain and the P1 subdomain, the P2 subdomain exhibits the most variable sequences, and the surface-exposed region contains the antigenic and histo-blood group antigen (HBGA) binding sites and is characterized by a high mutation frequency (Lindesmith et al., 2008). This is likely driven by host immune-selection pressure, which generates new epidemic strains with modified blockade epitopes and altered HBGA-binding properties (Schroten et al., 2016). NVs recognize as receptors HBGAs, which are complex carbohydrates present on red blood cells and the mucosal epithelium or as free antigens in biological fluids. The recognition of HBGAs by NVs is strain specific. Different residues in the P region may evolve in the recognition of different NV GII.4 strains.

The highly heterogeneous NVs are subdivided into ten genogroups (GI–GX), among which GI, GII, GIV, and GIX are associated with human diseases (Chhabra et al., 2019). GII.4 is the primary genotype responsible for global epidemics and 60–80% of outbreaks worldwide, with new variants emerging every 2 to 3 years (Yang et al., 2010); GII.4 has a faster mutation rate and evolution rate than other NVs (Bull et al., 2010). New epidemic and pandemic GII.4 variants appear and generally spread rapidly through populations and across continents. It appears that low cross-protection by specific anti-NV humoral immune responses play a major role in NV epidemics and pandemics (Debbink et al., 2013). It has been hypothesized that immunocompromised patients might be reservoirs where new NV variants emerge (Karst and Baric, 2015). Special attention should be paid to this population because of the extended nature of NV chronic infections and the reduced immuno pressure restricting viral mutation.

To evaluate NV evolution during chronic infection, we present a case of prolonged NV infection in a patient following hematopoietic stem cell transplantation (HSCT). We examined the intra-host NV populations and assessed immune-driven NV evolution in the chronically infected immunocompromised host and explored the strength of the positive selection for NV mutants.

## Materials and Methods

### Samples

Fifty stool samples were collected from 10 allo-HSCT recipients at different times from Beijing University First Hospital from February 2018 to March 2019. The stool specimens were transported on dry ice and stored at −80°C. Informed consent for fecal sample analysis was obtained from the patients or their legal guardians. The study protocol was approved by the Ethics Committee of the National Institute for Viral Disease Control and Prevention, China Center for Disease Control, according to Chinese ethics laws and regulations (IVDC2017-003).

### NV Genotyping, VP1 Amplification, and Quantitative PCR for GII.4 NV

Samples were diluted at a 1:10 ratio (w/v) with phosphate-buffered saline (PBS). Total RNA was extracted and the partial ORF2 gene was amplified by conventional RT-PCR for genotyping as described previously (Kojima et al., 2002). The three samples from the patient were subjected to amplification of the full capsid gene, as described previously (Vennema et al., 2002). The GII.4 NV viral loads in the samples were quantified by real-time PCR as reported previously (Kageyama et al., 2003).

### High-Throughput Sequencing

After dilution and filtration (0.45 and 0.22 μm membranes), total nucleic acids were extracted from the samples. Using the SuperScript^TM^ III Reverse Transcriptase (Invitrogen, United States), cDNA was synthesized, and were subjected to random PCR amplification using primers with unique barcodes. The PCR products were pooled for sequencing using the Illumina MiSeq platform (Illumina, San Diego, CA, United States). Metagenomic profiling of the shotgun datasets was carried out using the customized informatics pipeline VirusSeeker for computational identification of viral sequences (Zhao et al., 2017).

### Calculation of Genetic Diversity

All of the reads from each high-throughput sequencing sample were mapped to the P domain of the reference norovirus genome (accession no. MH229940) using Geneious (v.6.1.4). Diversity was quantified as the mean genetic distance calculated for all pairs of nt sequences using MEGA software (v.4.1). The rates of synonymous substitutions per synonymous site (dS) and of non-synonymous substitutions per non-synonymous site (dN) were calculated using the method of Nei and Gojobori with the Jukes-Cantor correction for multiple substitutions, using MEGA software (v.4.1). The dN/dS ratio is an indicator of the strength of the positive (>1) or negative (<1) selection pressure on a quasispecies.

### Selection Pressure on the VP1 Gene

The common residues of the epitopes and HBGA-binding sites of the P domain were annotated. Estimation of diversifying and purifying selection sites for the aligned VP1 sequences was performed using Datamonkey. The non-synonymous and synonymous substitution rates were calculated for each codon of VP1 using the mixed-effects model of evolution (MEME) and fast unbiased Bayesian approximation (FUBAR). The significance level was set at 0.05.

### Phylogenetic and Evolutionary Analyses

A phylogenetic analysis was performed using both the maximum-likelihood and maximum-parsimony methods in the PHYLIP package with 100 bootstrap replicates. To precisely estimate the substitution rate of VP1 in immunocompromised populations, a Bayesian Markov Chain Monte Carlo (MCMC) approach was implemented using BEAST software (v.1.8.2). jModelTest software (v.2.1.7) was used to identify the optimal evolutionary model. The results were computed and analyzed using Tracer v.1.6. The effective sample size values for the estimated parameters in the MCMC analyses were >200. Statistical uncertainty in the data was reflected by the 95% highest probability density values.

### Protein Modeling

Homodimeric P-domain capsid models were constructed for syh1, syh2, and syh3 using Phyre^1^ based on the template of NV GII.4 strain VA387 (c2obtA). The aa differences in the antigenic and HBGA-binding sites among syh1, syh2, syh3, and VA387 were plotted onto P-domain homology models to illustrate sequence variations.

## Results

### Case Description

Three of the fifty fecal samples were positive for NV GII.4. Interestingly, the three samples (syh1, syh2, and syh3) were collected at different times from the same patient. The patient was a 32-year-old female with anemia, transfusion dependency, febrile neutropenia, and thrombocytosis diagnosed as myelodysplastic syndrome, refractory anemia with excess blast 5–9% (MDS RAEB-I) in June 2016. An allo-HSCT was performed, in which the stem cells were from her HLA identical sibling. After the transplant, a long-term fever of up to 38.5°C developed gradually, accompanied by mild dyspnea. Conventional bronchoalveolar lavage fluid examinations such as bacterial and fungal culture, acid-fast stain of tubercle bacillus and PCR for Epstein–Barr virus and cytomegalovirus were negative. All anti-infection therapy was ineffective. Chronic lung graft versus host disease (GVHD) was suspected. Following oral administration of cortical hormone and tacrolimus, the dyspnea was relieved, and the body temperature returned to normal within 7 days. But 2 months later, the fever relapsed and persisted for more than 2 years accompanied by mild diarrhea (grade 1, CTCAE/NCI). Her stool was loose, without blood or pus. No bacterial, mycotic, or *Clostridium difficile* toxin A was detected in the stool. Three samples were collected on April 13, 2018 (syh1), November 19, 2018 (syh2), and March 25, 2019 (syh3). During this period, the patient had a fever of up to 37.8°C. She received oral cortical hormone (30 mg daily) and tacrolimus for 19 months to control the suspected chronic GVHD.

### High-Throughput Sequencing

The three NV-positive samples from the patient were subjected to high-throughput sequencing. A total of 67,770,700 reads was obtained. For syh1, 20,608 of 62,768 viral reads (32.8%) were identified as NV sequences, for syh2, 9057 of 76,531 viral reads (11.8%) were identified as NV sequences, while for syh3, 2372 of 82,009 viral reads (2.89%) were identified as NV sequences. Interestingly, herpesviruses were found in the syh1, syh2 and syh3, with 67, 276 and 63 reads, respectively, suggesting that herpesviruses may persistently exist in the host. Sample syh1 yielded a near full-length NV sequence and called 92.1% of the reference genome (MG214988.1, Henan, China) with 98.7% nt identity, lacking 480 and 150 bp at the 5′- and 3′-ends, respectively. The other two samples failed to yield a complete NV genome sequence (syh2, 79.0%; syh3, 78.8%), but the assembled sequences also showed the highest identities to the Henan strain, which belongs to the NV GII.4 Sydney 2012 cluster.

### Detection of Single-Nucleotide Variants

**FIGURE 1:**
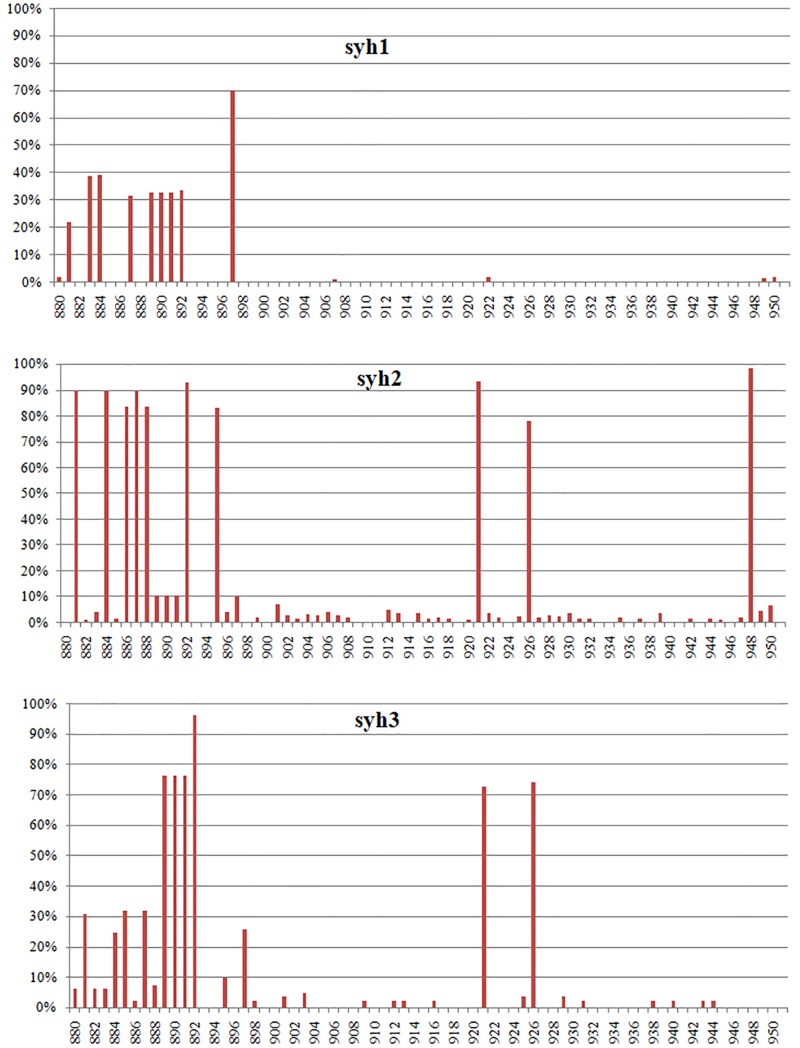
Distribution of NV SNV frequencies in the partial P region. Samples syh1 and syh2, and syh2 and syh3, were collected after 7 and 4 months, respectively. Only SNVs with frequencies >1% are included. The distributions of SNVs across the sequence in the three samples were similar, with most SNVs occurring at positions 880–895. *X*-axis, nt position; *Y*-axis, SNV frequency.

Single-nucleotide variants (SNVs) were identified in the high-throughput sequencing data to assess the intra-host genetic diversity. A short fragment (72 bp) in the P region was used for the analysis. We only called a SNV if it was observed in >1% of the reads to reduce false positives. Using this criterion, we detected different SNVs in the reads from the three samples. The first sample (syh1) had 14 SNVs, the second sample (syh2) had 51 SNVs, and the third sample (syh3) had 31 SNVs, indicating a high level of genetic diversity of NVs. Compared to the first sample, both the number and frequency of SNVs increased in the second and third samples. The lower number of SNVs in syh1 was not due to less sensitive detection, as it had a much higher average coverage than syh3, and comparable average coverage to syh2. The distributions of SNVs across the sequence in the three samples were similar (Figure 1), with most SNVs occurring at nt 880 to 895, which contains four amino acids (aas) that belong to epitope A and are reportedly targeted by antibodies blocking the binding of NV to human blood group antigens. The non-synonymous (dN) to synonymous (dS) substitution ratio was also determined (Table 1). The dN/dS ratios of the partial P domain for the three samples were >1, indicating strong positive selection in the patient.

**TABLE 1:** Basic information and NV readcoverage of the samples.

	**syh1**	**syh2**	**syh3**
Collection date	2018.04.13	2018.11.19	2019.03.25
Position (nt)^a^	5962–6035	5962–6035	5962–6035
Read No.^b^	480	587	81
dN	1.817	3.186	3.468
dS	0.634	0.978	1.589
dN/dS	**2.866**	**3.258**	**2.183**
SNVs No.	14	52	31

*No, number. ^a^Read no. refers to the reads covering the partial P domain. ^b^Position was compared to MG214988.1. Bold value means positive selection.*

### NV Quantification and VP1 Amplification

Specific real-time PCR was performed to quantify NV GII.4 in syh1, syh2, and syh3. The NV RNA concentrations were 2.46 × 10^6^, 1.47 × 10^6^, and 2.26 × 10^6^ genome copies/mL 10% (w/v) stool suspension, respectively, indicating low variability.

**FIGURE 2:**
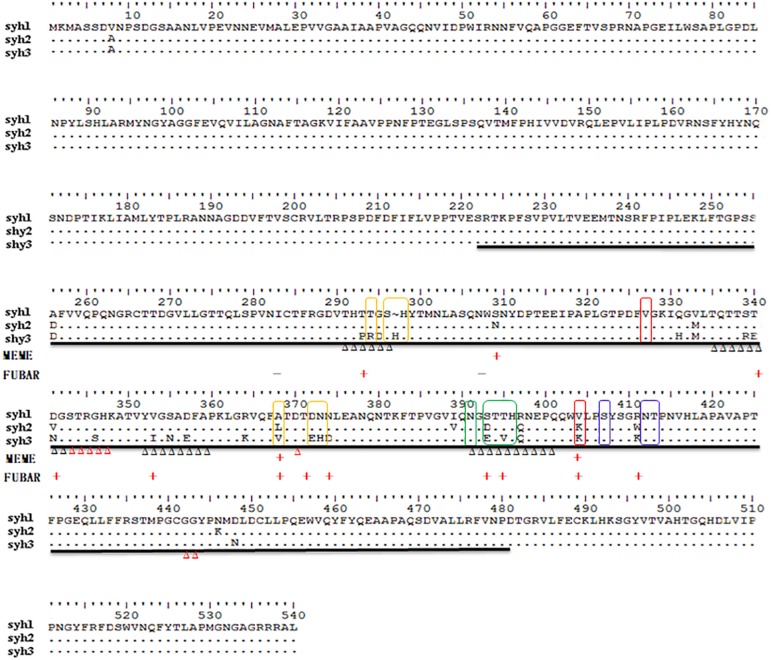
Analysis of capsid alignment of the haplotypes. Commonly defined epitopes are shown as colored frames. Orange, epitope A; green, epitope D; blue, epitope E; red, epitope F. HBGA-binding sites are marked by Δ, and red Δ are conserved aas constituting the GII conventional HBGA-binding interface. The positive and negative selection pressures determined by the Datamonkey algorithms are shown as red plus and black minus signs, respectively. Solid black-underlined sequence indicates the P domain of the capsid.

**FIGURE 3:**
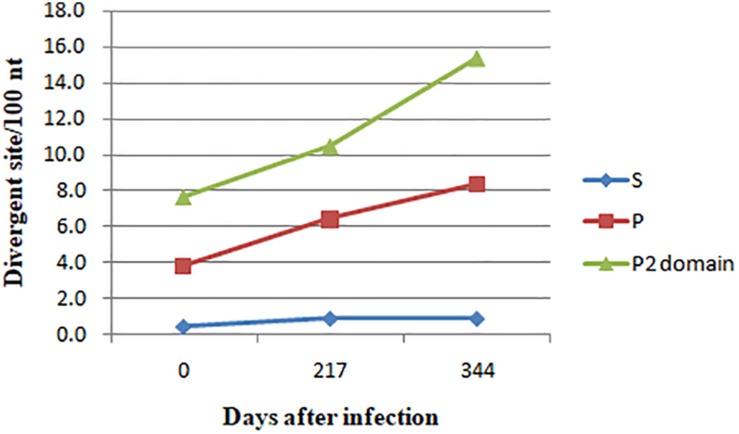
SNV accumulation in VP1 over time. The accumulation rate increased over time in the patient in the P domain and P2 subdomain, with the highest accumulation rate in the P2 subdomain.

The complete major capsid genes of the three NV-positive samples were amplified. syh1, syh2, and syh3 showed the best hit to the NV GII.4 HNZZ201707 strain (MH229940.1) from Henan, China, with nt identities of 98.34, 97.47, and 96.74%, respectively. The three sequences were deposited in GenBank under accession number MN764311-13. The deduced VP1 aa sequences of three haplotypes (539 aa in syh1 and syh2, 540 aa in syh3) were aligned and examined for aa changes. There were 29 non-conserved aa positions in the complete VP1 alignment, of which 28 were in the P domain and 18 were within defined epitopes and HBGA-binding sites. When compared with the first sample, there were more aa mutants in the third sample (26 aa differences) than in the second sample (12 aa differences) (Figure 2). The accumulation of mutations in VP1 was evaluated by SNP analysis of the S domain, P domain, and P2 subdomain. The accumulation rate increased over time in the patient. The highest accumulation rate of point mutations was in the P2 subdomain (15.4 variable sites per 100 nt). The accumulation rate was six-fold greater in the P domain than the S domain, with highest rates of 0.9 and 8.4, respectively (Figure 3).

To detect the codons that provide stronger evidence of purifying or diversifying selection, the coding sequences of these haplotypes were examined using Datamonkey. All negative and positive selection happened in the P-domain, negative selection was defined at 2 sites (by at least one of the two methods, black plus sign), while positive selection was defined at 11 sites (by at least one of the two methods, red plus sign) (Figure 2), among which sites 368 and 404 had the strongest support (positive by both the MEME and FUBAR methods). Five (sites 368, 372, 393, 395, and 404) of the twelve positive selection sites were part of known epitopes A, D, E, and F, while six (sites 293, 340, 341, 353, 393, and 395) were a part of HBGA-binding sites. Positive sites 309, 374, and 411 in the P domain were not part of any epitope sites or HBGA-binding regions.

### Phylogenetic Analysis

**FIGURE 4:**
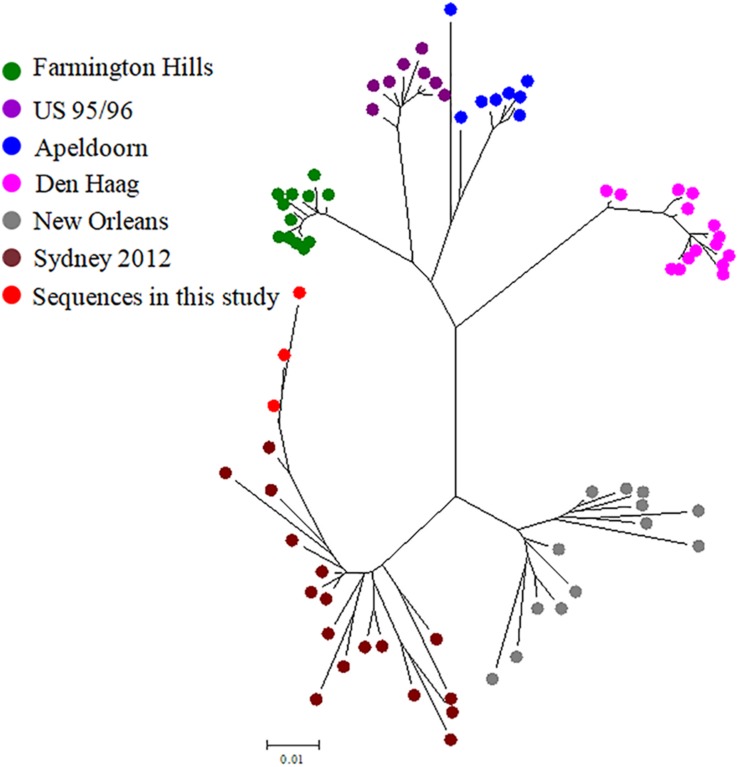
Maximum-likelihood phylogenetic tree of the VP1 nt sequences of the patient compared to representative related NV GII.4 VP1 sequences in GenBank. Reference sequences are indicated by colors; red nodes represent sequences from the patient in this study. The three VP1 sequences belong to Sydney 2012, and were closest to the Henan strain from China.

To study within-host virus evolution, the VP1 sequences from immunocompromised patients were compared to GenBank reference sequences by maximum-parsimony analysis. The three sequences from the patient were closely related to the HNZZ201707 strain (Figure 4), which was detected in 2017, Henan, China. They formed a separate branch in the Sydney 2012 cluster.

### Modeling of HBGA Binding Region

**FIGURE 5:**
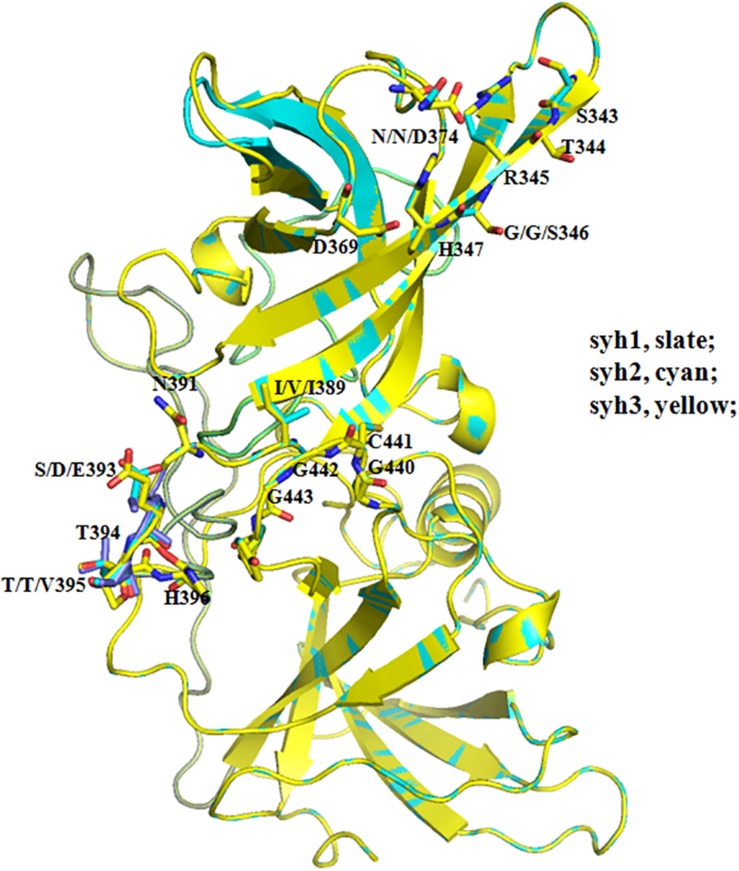
Structural comparisons of the HBGA-binding site among the three haplotypes. Slate, syh1; cyan, syh2; and yellow, shy3. Most of the region below the HBGA pocket was unchanged; however, site 393–396 had marked differences and site 374 had mild changes among the three samples.

Comparing the sequences of syh1, syh2, and syh3, there were notable aa substitutions in the receptor-binding pocket sites and sites 293, 340, 341, 353, 393, and 395 were under positive selection (Figure 2). Homology models of the three samples were constructed to examine the influence of changes at the receptor-binding pocket sites (Figure 5). Most of the region directly below the HBGA pocket remained unchanged; however, site 393–396 had marked differences and site 374 had small differences among the three samples.

### Homology Model of the P Domains

**FIGURE 6:**
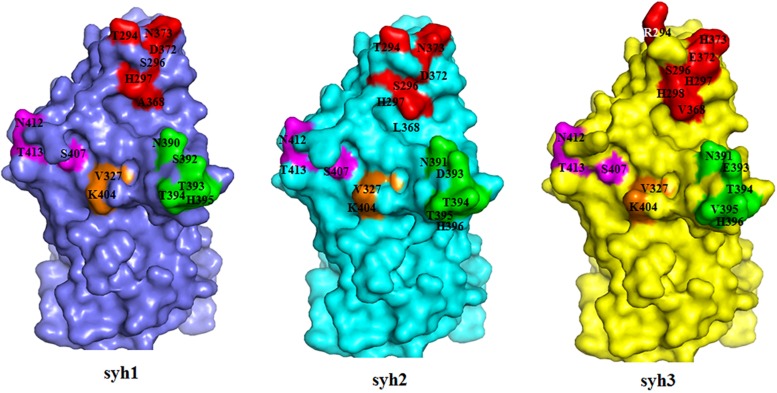
Within-host aa changes of VP1 blockade epitope A, D, E, and F among the three samples. Red, epitope A; green, epitope D; purple, epitope E; and orange, epitope F. The spatial conformation of the P1 domain was unchanged, but epitopes A and D and their adjacent areas in the P2 domain were markedly different among syh1, syh2, and syh3.

A structure of the NV VA387 P domain has been generated and used to locate within-host aa changes in the three samples (syh1, syh2, and syh3) to study the effect of within-host evolution. The aa positions of the major blockade epitopes (A, D, E, and F) for GII.4 were plotted onto P-domain homology models for the patient over time (Figure 6). The spatial conformation of the P1 domain remained unchanged, but there were many differences in the P2 domain among syh1, syh2, and syh3. Compared with the conformation difference between syh2 and syh1, the difference between syh3 and syh1 was greater, especially in the major epitope sites and their adjacent regions. Because there was no aa mutation in epitopes E and F among the three samples, there was little difference in the spatial conformation between these two epitopes, but there was an obvious conformation difference in the region near epitope E. Because of the aa change in epitope D, the spatial conformation was different in the three samples. Epitope A was the most variable, and that of syh3 showed marked differences in spatial conformation compared to syh1 and syh2.

## Discussion

Despite its high population seroprevalence, GII.4 NV strains have circulated worldwide for decades, and new strains emerge and replace ancestral strains. Different GII.4 NVs exhibit different ligand-binding properties and antigenicity (de Rougemont et al., 2011; Lindesmith et al., 2012a). New GII.4 NV strain emergence is associated with changes in antibody blockade epitopes, and it is assumed that GII.4 NV persistence in the human population is driven by viral evolution that results in antigenic drift and changes in binding characteristics to escape herd immunity. NV infections in immunocompromised patients after transplantation are regarded as chronic symptomatic infections. It was found that numerous mutations in the capsid did not alter the HBGA binding profiles, and chronically infected patients might not generate novel variants that cause outbreaks (Doerflinger et al., 2017). However, there was more evidence that intrahost GII.4 evolution can lead to antigenic epitopes change, which may produce potential successive outbreak strains (Debbink et al., 2014; Mai et al., 2016; Steyer et al., 2018). Our data provided insight into the extensive genetic diversity and evolution of NVs in a patient following allo-HSCT, and suggested that novel variants of NV GII.4 with HBGA and antigenic site changes were produced; chronically infected cases may serve as a potential reservoir for novel NVs.

One of 10 patients (1%) or 3 of 50 (0.6%) fecal samples were positive for NVs, lower than a previously report (10%) in hematological malignancies (Bok et al., 2016). This may be related to the short time of sample collection and the small number of samples collected in this study. Interestingly, the three NV-positive samples were from the same patient. Although the patient had been using anti-rejection drugs after transplantation, symptoms of mild diarrhea and long-term fever persisted. No bacterial, mycotic, or *C*. *difficile* toxin was detected, and no other common diarrhea-related virus was found in the stool. Although the blood was negative for NV (data not shown), the quantitative results showed that the concentration of NV in feces was stable at 10^6^ copies/mL 10% (w/v) stool suspension, which was lower than that in acute infection and comparable to that in chronic infections (Ludwig et al., 2008; Steyer et al., 2018). We cannot determine whether the patient’s fever and diarrhea were caused by NV. However, it can be inferred that the virus adapted to host immunity so that it can be stable in the host; on the other hand, the moderate immunity of the host provided selective pressure to the NVs and could generate new variants, which had been reported in previous studies (Debbink et al., 2014).

The NVs reported in this study were most similar to the strain reported in Henan, China in 2017, followed by that in Sydney in 2012. Sequence analysis of the P domain showed that the NV population in the patient was highly heterogeneous at the nt level and encompassed a large number of NV quasispecies. The dN/dS ratios of the S and P domains were >1, indicating strong positive selection and suggesting that a large number of NV quasispecies were present in the host. A previous study showed many positive selection sites in the capsid protein of an enterovirus with strong antigenicity (Zhang and Lu, 2010). We speculate that the presence of positive selection sites in the capsid protein of the virus may explain the viral divergence observed in allo-HSCT patients.

The number of mutations in the three samples increased over time, and the mutation rate was not equally distributed throughout the gene. It was markedly greater in the P region than in other regions, especially the P2 subdomain, which includes antibody blockade epitopes and HBGA-binding sites. Several aas in the sites are under strong positive selection pressure. Of the 12 positive selection sites, 9 were located at HBGA-binding or antigenic sites. As reported previously, this can result in the generation of new epidemic NV variants (Bull et al., 2012; Lindesmith et al., 2013). Six sites under positive selection were located in HBGA-binding sites, of which residue 293 might influence epitope A structure as it was in the 8 Å expanded area (Lindesmith et al., 2012a). Overall, the conformation of HBGA-binding sites showed little change among the three samples; however, a significant difference at 393–396 was observed. Sites 343–347, 374, and 442–443 represent eight conserved aas that constitute the GII conventional HBGA-binding interface (Liu et al., 2015); however, sites 346 and 374 were mutated in syh3, which slightly altered the conformation. These results suggested that capsids of chronically infecting NVs interact with HBGAs differently than epidemic GII.4 NVs.

Epitope A is a known major blockade epitope, consisting of aa residues 294, 296, 297, 298, 368, and 372, of which 296 was reportedly completely conserved during intra-host NV evolution (Bull et al., 2012), and was conserved (Ser296) in the samples in this study. While sites 368 and 372 in epitope A were under strong positive selection, in particular, site 368 differed among the three samples. As reported previously, changes at 12 sites in the P2 region might contribute to an escape phenotype, among which sites 368 and 372 play direct roles in variation (Lindesmith et al., 2012a). In this study, the conformation of epitope A in syh3 was markedly different from those in syh1 and syh2, indicating the future appearance of a new phenotype(s).

Sites 393 and 395 of epitope D and site 404 of epitope F were also under positive selection. Epitope D is an evolving blockade epitope and residues 393 and 395 can directly impact Lewis HBGA binding of GII.4 strains (Shanker et al., 2011; Lindesmith et al., 2013), so mutation of sites 393 and 395 may change viral binding characteristics. The other sites in epitopes D and F were conserved in the present study. Mouse monoclonal antibodies have confirmed epitope E to be GII.4 2002 Farmington Hills-specific blockade epitope (Lindesmith et al., 2012b). In this study, all common residues of epitope E were conserved in the evolving haplotypes, indicating that changes in other aas lead to changes in the spatial structure of the adjacent region of epitope E, and so the antigenicity in this region is not affected.

A phylogenetic analysis of NV haplotypes based on the capsid nt sequences showed that the three variants belong to the Sydney 2012 lineage, and were closest to the HNZZ201707 strain, which was identified in China in 2017. Therefore, the patient may have been exposed to circulating NV strains. However, the first sample, syh1, showed only 98.34% nt identity to HNZZ201707, suggesting that the patient was infected some time before the initial sampling. With the accumulation of mutations, the distance between the variants and the HNZZ201707 strain increased over time within the host. Therefore, NVs may evolve into new strains quite different from known NV GII.4 strains.

Overall, our data showed that GII.4 NV can cause prolonged infection in immunocompromised patients, and the immunodeficient NV RNA population in the host with positively selected antigenic and HBGA sites suggested immunocompromised patients to be potential reservoirs for novel NV strains that escape herd immunity. Because of the typically prolonged hospitalization and lack of management after discharge from hospital of immunocompromised patients, there is a marked risk of direct or indirect contact with other susceptible patients and even healthy individuals. Further study is needed to characterize the infectious capacity of NV variants in the study population.

## Data Availability Statement

The raw data supporting the conclusions of this article will be made available by the authors, without undue reservation, to any qualified researcher.

## Ethics Statement

The studies involving human participants were reviewed and approved by the Ethics Committee of the National Institute for Viral Disease Control and Prevention, China Center for Disease Control. Written informed consent for participation was not required for this study in accordance with the national legislation and the institutional requirements.

## Author Contributions

JY, ZD, and YD designed the study. JY, ZL, QZ, and KG collected the samples and analyzed the data. JY, XS, and ZL did the experiment. JY and ZL wrote the manuscript. YD and ZD reviewed the manuscript. All authors have read and approved the manuscript to publish.

## Conflict of Interest

The authors declare that the research was conducted in the absence of any commercial or financial relationships that could be construed as a potential conflict of interest.

## Footnotes

1 http://www.sbg.bio.ic.ac.uk/phyre2/html/page.cgi?id=index
